# Persistent level 1 hypoglycemia due to hypothyroidism and underlying Neurofibromatosis type 1

**DOI:** 10.1002/ccr3.7278

**Published:** 2023-05-01

**Authors:** Hoang Nguyen, Quynh Thu Nguyen, Zeyar Thet, Thanh D. Hoang

**Affiliations:** ^1^ Department of Medicine Wyckoff Heights Medical Center Brooklyn New York USA; ^2^ Department of Pediatrics Brookdale University Medical Center Brooklyn New York USA; ^3^ Department of Medicine, Division of Endocrinology Walter Reed National Military Medical Center Bethesda Maryland USA

**Keywords:** neurofibromatosis, NF1, persistent hypoglycemia

## Abstract

**Key Clinical Message:**

Hypoglycemia in non‐diabetic patients is rare and may be due to various etiologies. It is important to recognize hypoglycemia early and appropriately manage hypoglycemia in patients with neurofibromatosis 1 and hypothyroidism.

**Abstract:**

Non‐diabetic hypoglycemia is not common and can be seen in certain conditions like Neurofibromatosis type 1 (NF1). We report a rare case of 66‐year‐old man with hypothyroidism and NF1 who developed a persistent level 1 hypoglycemia.

## Graphical abstract

Hypoglycemia is rare in non‐diabetic patients. Hypoglycemia can be seen in patients with hypothyroidism and neurofibromatosis type 1.

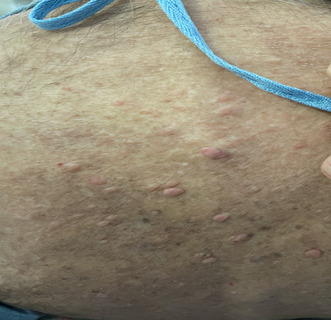

## 1 INTRODUCTION

Neurofibromatosis (NF1), also well‐known as von Recklinghausen's disease, is an autosomal dominant disease which affects multiple organs in the body.^1^ The incidence is approximately 1 per 2500–3000 individuals worldwide, independent of gender and ethnicity.^2^ The diagnosis of the disease based mainly on clinical history taking and physical examination. According to NIH criteria,^3^ a patient who is diagnosed with NF1 when meeting 2 out of 7 clinical features: first degree relative of NF1, ≥2 cutaneous or subcutaneous neurofibromas, ≥6 café‐au‐lait patches, axillary or groin freckling, ≥2 iris Lisch nodules, optic pathway glioma, and distinctive bony dysplasia.^4^

Hypoglycemia is a well‐known presentation in diabetic patients but is rare in non‐diabetic populations. There are several non‐diabetic causes for hypoglycemia such as medications, critical illnesses, hormone deficiency, adrenal insufficiency, and endogenous hyperinsulinism.^4^, ^5^ We presented a case of a newly diagnosed NF1 non‐diabetic patient with chronic fatigue and persistent level 1 hypoglycemia.

## 2 CASE REPORT

A 66‐year‐old man initially presented to the emergency room because of weakness and altered mental status. Vital signs showed bradycardia (57 bpm), hypothermia (32°C), blood pressure (143/90 mmHg), and respiratory rate 18 per minute with SpO2 99% on room air. Physical examination revealed alert and oriented × 2 with self and place, supple neck, no focal neurological deficits, and no pitting edema. Head CT scan without contrast and head MRI were done without any acute intracranial process. His past medical history is significant for Parkinson's disease (diagnosed in 2010) for which he was taking Carbidopa‐Levodopa 25–100 mg, and ropinirole XL 4 mg, asymptomatic positive COVID‐19 1 month prior.

Initial laboratory tests indicated WBC 5.1 k/uL (normal 4.5–11.0 × 10^9^/L), FT4 0.4 ng/dL (normal 0.7–1.8); high TSH 13.4 mIU/L (normal 0.5–5.0), blood glucose 69 mg/dL (normal 72–99), normal metabolic panel, and HbA1C 5.1%.

**FIGURE 1:**
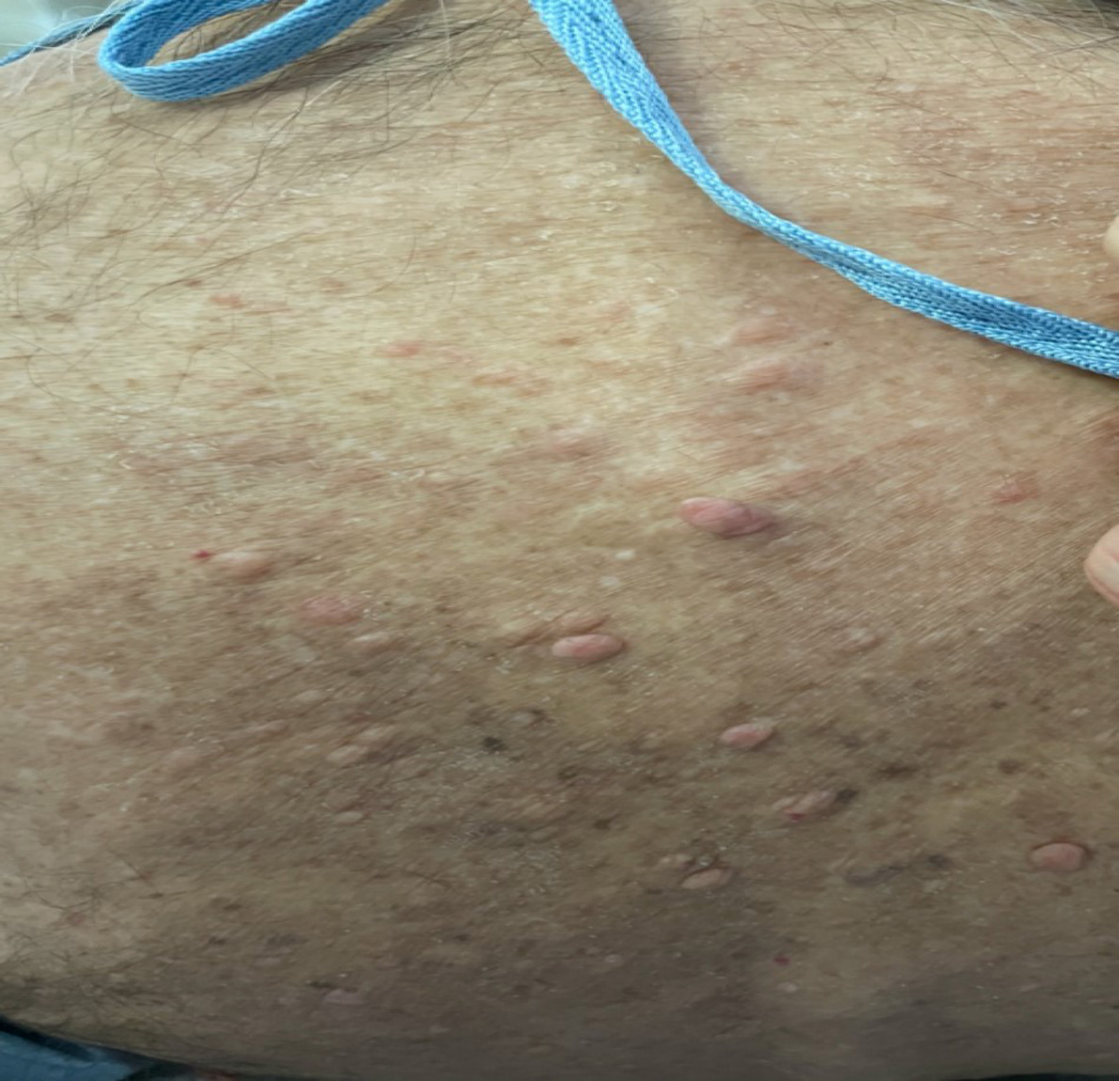
Cutaneous fibromas on the back.

**FIGURE 2:**
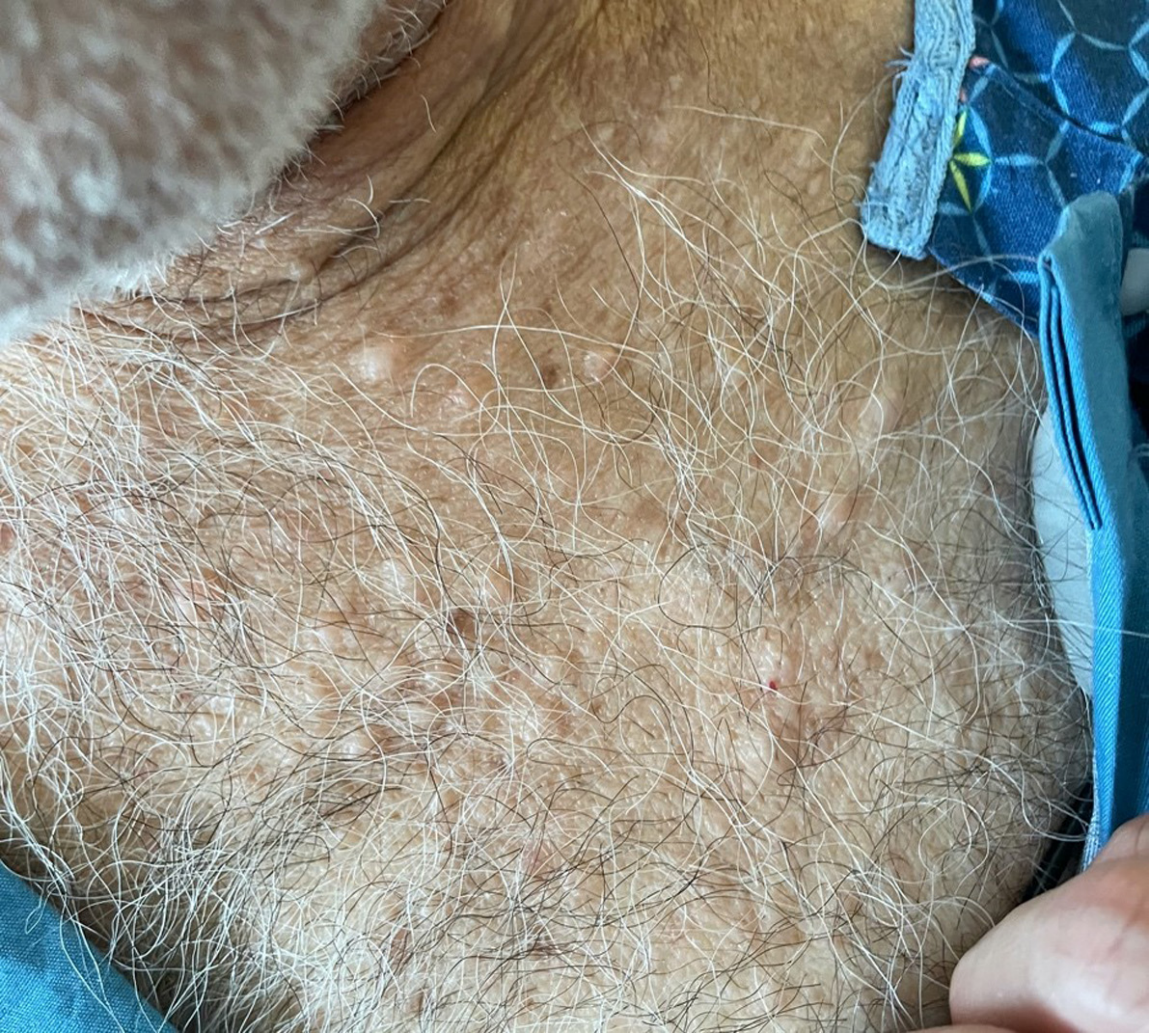
Cutaneous fibromas on the chest.

**FIGURE 3:**
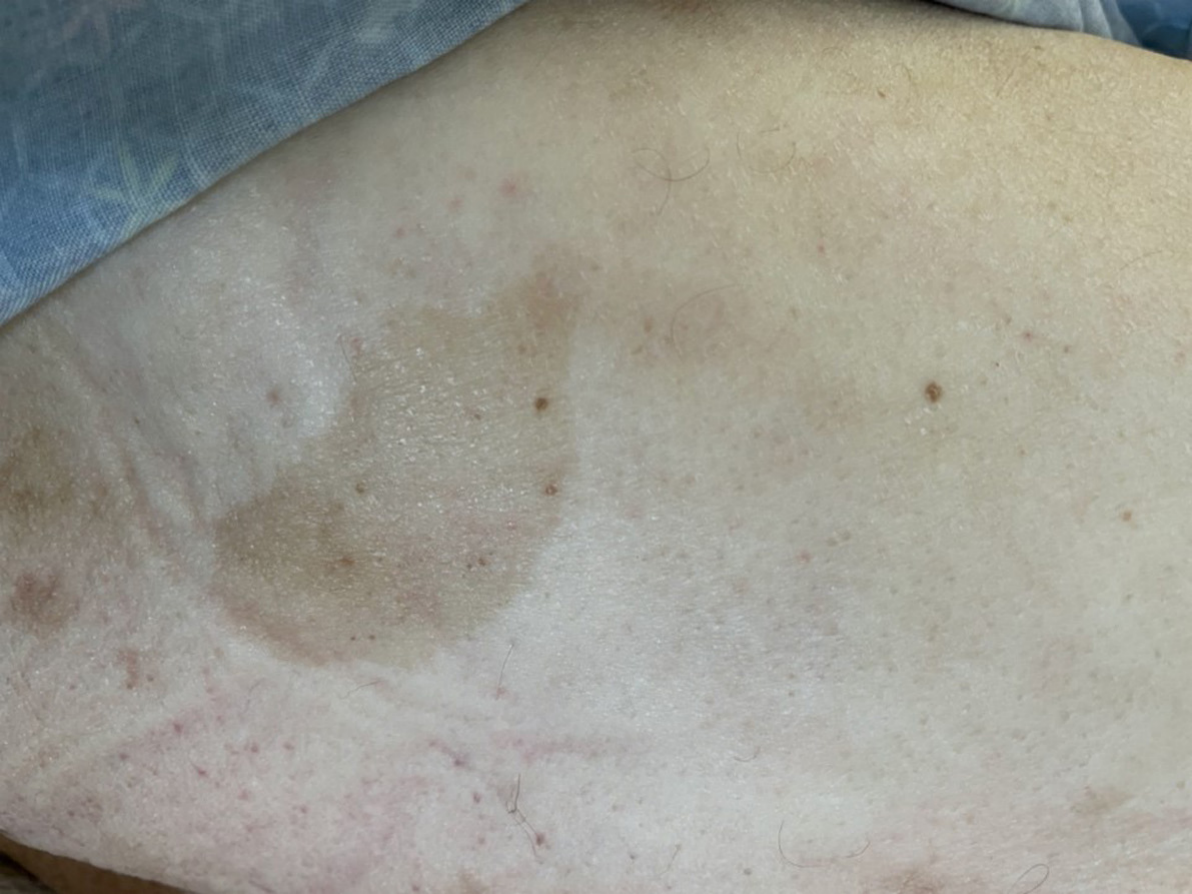
Café‐au‐lait macule on the right thigh.

Presumptive diagnosis was metabolic encephalopathy secondary to suspected hypothyroidism and hypoglycemia. Patient was treated with Dextrose 50% and transferred to the Medicine floor for further evaluation and treatment. On the Medicine floor, he was treated with levothyroxine 25mcg daily orally. Further examination revealed many cutaneous fibromas all over his body, especially on his back and chest (Figure 1 and Figure 2), along with cafe‐au‐lait patches (Figure 3) and groin freckles that he has since childhood. Family history showed that his sister also has similar signs but has not yet been diagnosed. Patient was diagnosed with Neurofibromatosis type 1 according to clinical criteria.

During the next 3 days, his heart rate and temperature returned to normal range. However, he still feels tired. His morning fasting blood glucose levels were mildly low: 69 mg/dL, 90 mg/dL, 77 mg/dL, 67 mg/dL. Patient underwent further evaluation for hypoglycemia. Abdomen and chest CT‐scan with contrast did not show any tumors. Anti‐thyroglobulin Ab <1 IU/mL, anti‐peroxidase ab <1 IU/mL; insulin ab <0.4 U/mL; IGF‐1 50 ng/dL; IGF‐binding protein‐3 low 1.7 mg/L, IGF 2: N/A, Insulin level: 10 mcU/mL, C‐peptide 1.1 ng/mL, serum cortisol level: 15 mcg/dL. Since he did not show critical symptoms, he was instructed to eat small meals between 3 regular meals and monitor blood glucose at home. He was discharged with close follow‐up.

## 3 DISCUSSION

There may be a combination etiology that make our patient has persistent hypoglycemia: NF‐1 and hypothyroidism. Hypothyroidism is associated with various hormonal biochemical and nervous system abnormalities, which can lead to hypoglycemia, such as reduced basal and stimulated growth hormone or cortisol levels, blunted hypothalamo‐pituitary‐adrenal response, reduced glucogenesis, impaired glycogenolysis, reduced glucagon secretion, and slowing of insulin clearance.^6^, ^7^, ^8^ There are well established associations between hypoglycemia condition and hypothyroidism. In our case, when the patient presented with hypothyroid signs and symptoms, the first impression was hypoglycemia due to hypothyroidism. However, after hypothyroidism treatment, his condition improved except the hypoglycemia that was persistent despite extensive treatment. At that moment, given patient clinical picture, the other conditions were investigated (including insulinoma which was excluded by imagines studies and laboratory) and we suspected likely NF1 etiology on top of hypothyroidism which made his hypoglycemia condition more severe and hard to manage.

Neurofibromatosis is a very rare disease and mostly diagnosed clinically. Beside classical features, there are various conditions that we need to be aware of, such as pheochromocytoma, carcinoid, and osteoporosis.^9^ Recently, Martins et al found out that there is a lower level of fasting blood glucose (FBG) and a lower prevalence of high FBG level in NF1 patients compared with non‐NF1 patients. Median FBG level in the NF1 patients is 86 mg/dL (ranging from 56 to 127 mg/dL) which was significantly lower than that in the non‐NF1 patients whose mean FBG is 102 mg/dL (ranging from 85 to 146 mg/dL).^10^, ^11^ Additionally, Madubata et al. also showed that a significant lower rate of diabetes‐related healthcare claims among NF1 patients compared with Non‐NF1 population.^12^

In 2020, Marinescu et al reported an unusual hypoglycemia/ hyperinsulinism condition in a NF‐1 infant.^13^ One hypothesis is that there may be lower levels of resistin, visfatin, and leptin but a higher level of adiponectin in NF1 patients. This feature might reduce insulin resistance, lower FBG level and reduce developing of type 2 DM. Another hypothesis could be due to the secretion of insulin‐like growth factor 2 (IGF2) by neurofibromas.^10^, ^11^, ^12^ In addition, higher IGF2 level increases peripheral glucose utilization, diminishes liver's glucose production, and causes hypoglycemia.^14^ Unfortunately, we did not obtain IFG2 in our patient.

The association between IGF‐1, IGF‐binding protein level, and insulin resistance has been showed in several researches.^15^, ^16^, ^17^, ^18^ While both high and low IGF‐1 level may be associated with glucose intolerance and insulin resistance (U‐shaped effect),^15^, ^18^ there are positive correlation between IGFBP3 (IGF‐binding protein 3) and glucose intolerance/insulin resistance.^17^, ^19^, ^20^ Our case has normal IGF‐1 level and low IGF‐binding protein 3. It may indicate that the patient is insulin sensitive.

Currently, there is no recommendation regarding treatment hypoglycemia in NF‐1 patient. Our patient did not have critical signs and symptoms of hypoglycemia. Hence, he was asked to monitor his glucose frequently at home, take several small portions meals daily, and to always carry glucose tablets with him. Patient declined any severe hypoglycemic episodes.

## 4 CONCLUSION

Hypoglycemia in non‐diabetes mellitus patients is quite rare and may be due to various etiologies. Persistent hypoglycemia in NF1 patient is not well defined in the current literature. Our case highlights the importance of early recognition and appropriate management of persistent level 1 hypoglycemia in patients with NF1 and hypothyroidism. Further studies are needed to clarify the association.

## AUTHOR CONTRIBUTIONS

**Hoang Nguyen:** Conceptualization; writing – original draft. **Quynh Thu Nguyen:** Conceptualization; resources; writing – review and editing. **Zeyar Thet:** Conceptualization; formal analysis; writing – review and editing. **Thanh D Hoang:** Conceptualization; resources; supervision; writing – review and editing.

## FUNDING INFORMATION

None.

## CONFLICT OF INTEREST STATEMENT

None to declare.

## ETHICS STATEMENT

The manuscript has been reviewed and approved by the IRB and Public Affairs Office.

## CONSENT STATEMENT

The authors have confirmed that patient consent has been signed and collected in accordance with the journal's patient consent policy.

## Data Availability

Not applicable.
